# Novel Imaging Enhancements in Capsule Endoscopy

**DOI:** 10.1155/2013/304723

**Published:** 2013-06-26

**Authors:** Mostafa Ibrahim, Andre Van Gossum

**Affiliations:** Erasme Hospital, Université Libre de Bruxelles, Brussels, Belgium

## Abstract

Video capsule endoscopy that was launched 10 years ago has become a first-line procedure for examining the small bowel. The most common indications for capsule endoscopy are obscure gastrointestinal bleeding, Crohn's disease, polyposis syndromes, and evaluation of patients with complicated celiac disease. The ideal capsule should improve the quality of the image and have a faster frame rate than the currently available one. There should be a therapeutic capsule capable of performing a biopsy, aspirating fluid, delivering drugs, and measuring the motility of the small bowel wall. Another major leap forward would be the capability of remote control of capsule's movement in order to navigate it to reach designated anatomical areas for carrying out a variety of therapeutic options. Technology for improving the capability of the future generation capsules almost within grasp and it would not be surprising to witness the realization of these giant steps within the coming decade. In this review we will focus on the current clinical applications of capsule endoscopy for imaging of the small bowel and colon and will additionally give an outlook on future concepts and developments of capsule endoscopy.

## 1. Introduction

Small bowel capsule endoscopy (SBCE) was introduced 13 years ago. To date, multiple CE systems from different companies are available. Currently the Given M2A Video Capsule System (Given Imaging Ltd., Yokneam, Israel), the Olympus EndoCapsule (Olympus, Tokyo, Japan), and the MiroCam (Intromedic, Seoul, Korea) are FDA and CE approved. Capsule systems are available for examination of the esophagus, small bowel, and colon. 

Capsule endoscopy is easily ingested and swallowed by most individuals, but also, a capsule-loading device (AdvanCE, US Endoscopy, Mentor, OH, USA) is available to directly deliver the capsule into the stomach or duodenum. The disposable device is a 2.5 mm single-sheathed device that is first preloaded through the working channel of a standard endoscope. The main indications for SBCE include obscure GI bleeding, Crohn's disease, and celiac disease [1].

## 2. Patient Preparation

There are several accepted preparation methods for SBCE for small bowel CE, which include fasting since the day before, clear liquid diet, the ingestion of 2–4 liters of polyethylene glycol solution. In addition, some experts recommend the use of simethicone before the ingestion of the capsule to reduce intraluminal foam and bubbles [2].

## 3. Indications

### 3.1. Obscure Gastrointestinal Bleeding

Many reports have shown a statistically significant increased diagnostic yield of SBCE over push enteroscopy and other modalities including magnetic resonance enteroclysis in the range between 39 and 90% [3] and a similar yield to balloon-assisted enteroscopy [4]. 

A pooled analysis of 7 prospective studies showed a CE yield of 71% for identification of a bleeding source compared to push enteroscopy [5]. When comparing CE with intraoperative enteroscopy, the sensitivity, specificity positive, and negative predictive value of CE was 95, 75, 95, and 86%, respectively [6]. 

A negative capsule endoscopy study in patients with obscure bleeding is associated with low rate of recurrent bleeding. Apart from active bleeding, there are no other helpful features to determine that arteriovenous malformations are the cause of bleeding [7]. 

The diagnostic yield is influenced by the timing of the examination, and a recent study investigated the role of early CE in the diagnosis of patients with obscure GI bleeding. They assessed ninety patients with obscure GI bleeding, and they showed that the duration between bleeding and CE was shorter for patients with angioectasia than for those with other abnormalities. The authors concluded that earlier timing of CE achieved a higher diagnostic yield for patients with obscure GI bleeding and consequently resulted in a higher intervention rate [8].

### 3.2. Crohn's Disease

Various studies have shown the potential of SBCE for diagnosis of Crohn's disease [9–13]; SBCE can identify mucosal lesions that are compatible with CD in some patients in whom conventional endoscopic and small bowel radiographic imaging modalities have been nondiagnostic, but the diagnosis of Crohn's disease should not be based on the appearance at the capsule endoscopy alone. However, SBCE is better than small bowel follow-through, enteroclysis, computed tomography (CT) enterography, or MRI enteroclysis for detecting mucosal lesions related to CD [14]. 

A recent multicenter, double blind, prospective, controlled study of SBCE videos from 62 consecutive patients with isolated small-bowel Crohn's disease was designed to evaluate three main parameters of Crohn's disease: inflammation (*A*), extent of disease (*B*), and stricture (*C*), in both the proximal and distal segments of the small bowel. 

The Capsule Endoscopy Crohn's Disease Activity Index (CECDAI or Niv score) was devised to measure mucosal disease activity using video capsule endoscopy (VCE).

The final score was calculated by adding the two segmental scores: CECDAI = ([*A*1 × *B*1] + *C*1) + ([*A*2 × *B*2] + *C*2). Each examiner in every site interpreted 6–10 videos and calculated the CECDAI. 

The authors showed that the cecum was reached in 72% and 86% of examinations, and proximal small-bowel involvement was found in 56% and 62% of the patients, according to the site investigators and principal investigator, respectively. Significant correlation was demonstrated between the calculation of the CECDAI by the individual site investigators and that performed by the principal investigator. They showed that also overall correlation between endoscopists from the different study centers was good, with *r* = 0.767 (range 0.717–0.985; Kappa 0.66; *P* < 0.001). There was no correlation between the CECDAI and the Crohn's Disease Activity Index or the Inflammatory Bowel Disease Quality of Life Questionnaire [15]. 

In these patients, the risk of SBCE retention is increased, particularly in these with known intestinal stenosis. A Agile patency capsule may reduce the risk of retention. The Agile patency capsule has the same size as the SBVCE. It has cellophane walls that are filled with lactose (mixed with barium) and surround a radio-frequency identification tag (RFID). When retained in a fluid filled environment, the core of the patency capsule dissolves after approximately 40 hours, allowing the insoluble outer membrane to collapse and pass. The physician can determine the presence of the patency capsule in the body of the patient using the scanner. The Agile capsule is expected to eliminate risk of capsule retention in patients with known intestinal strictures who undergo capsule endoscopy [16].

### 3.3. Other Indications for SBCE

#### 3.3.1. Intestinal Tumors

SBCE has more than doubled the rate of diagnosing small bowel tumors from around 3 to 6%, most of the tumors are found in patients undergoing the exam for OGIB, and 50–60% are of malignant nature [17]. 

#### 3.3.2. Celiac Disease

A recent published meta-analysis described an overall pooled sensitivity of 89% (95% CI 82–94) and specificity of 95% (95% CI 89–98) for CE in celiac disease [18], on the contrary the gold standard for Celiac disease is to date the histopathological assessment. 

## 4. Novel Imaging Enhancements

### 4.1. Fujinon Intelligent Chromoendoscopy-Assisted Capsule Endoscopy

The FICE technology decomposes images by three specific wavelengths (red, green, and blue) and then directly reconstructs the images with enhanced surface contrast. The FICE software has recently been incorporated into the new RAPID 6.0 video CE workstation (Given Imaging Ltd, Yokneam, Israel). With this innovation, the examiner can easily select between conventional images and images reconstructed under three different FICE settings by the click of an icon in the Rapid Reader software for optimal mucosal visualization. Capsule endoscopy with flexible spectral imaging color enhancement (CE-FICE) has been reported to improve the visualization of small-bowel lesions; however, its clinical usefulness is still not established. 

A recent study evaluated whether CE-FICE contributes to improve the detectability of small-bowel lesions. The authors examined a total of 60 angioectasias; CE trainees identified 26 by conventional CE. The authors concluded that FICE settings 1 and 2 significantly improved the detectability of angioectasia (*P* = 0.0017 and *P* = 0.014, resp.) and erosions/ulcerations (*P* = 0.0012 and *P* = 0.0094, resp.). Although the detectability of small-bowel lesions by conventional CE (*P* = 0.020) and under FICE setting 2 (*P* = 0.0023) was reduced by the presence of bile pigments, that under FICE setting 1 was not affected (*P* = 0.59) [19]. 

Another study assessed the usefulness of flexible spectral imaging color enhancement (FICE) for the detection of angiodysplasia. The authors assessed the accumulated SBCE data of 50 patients with angiodysplasia that were randomly assigned to 2 equally sized groups of conventional reading and FICE reading. One experienced doctor analyzed them for the first time in a quick-view mode, and the mean reading time, sensitivity, and specificity for detecting angiodysplasia by each method were evaluated for comparisons including suspected blood indicator. The authors showed that the mean reading time was 14 min for both conventional reading and FICE reading. The two previews of angiodysplasia were significantly superior to the function of suspected blood indicator (*P* < 0.01). The sensitivity and specificity of conventional reading for detecting angiodysplasia were 80% and 100%, respectively. Those of FICE reading were 91% and 86%, respectively. FICE reading was superior in term of sensitivity, while it resulted in more false positive lesion findings and lower specificity. However, such false-positive findings by FICE reading can be correctly identified at a glance by converting the image to conventional mode [20]. 

One of the earlier studies assessing FICE examined P0, P1, and P2 lesions (nonpathological, intermediate bleed potential, and high bleed potential) in 60 patients, overall 157 lesions were diagnosed using FICE as compared to 114 with white light SBCE (*P* = 0.15). For P2 lesions, the sensitivity was 94% versus 97%, and specificity was 95% versus 96% for FICE and white light, respectively. Five (P2 lesions) out of 55 arteriovenous malformations could be better characterized by FICE as compared to white light SBCE. Significantly more P0 lesions were diagnosed when FICE was used as compared to white light (39 versus 8, *P* < 0.001). The author concluded that FICE was not better than white light for diagnosing and characterizing significant lesions on SBCE for OGIB [21]. Another negative study conducted on 27 patients to check the usefulness of blue mode (BM) review in Lewis score (LS) calculation, by comparing it with respective LS results obtained by white light (WL) small-bowel capsule endoscopy (SBCE) review and mucosal inflammation as reflected by faecal calprotectin (FC) levels, considered as “gold standard” for the study. 

LS was created in four separate steps. First, parameters and descriptors of inflammatory change were identified. Secondly, blinded readers prospectively graded the presence or absence of each parameter on de-identified videos and graded a perceived global assessment of overall severity. Thirdly, the individual parameters and descriptors were ranked in order of severity. Fourthly, values for each parameter were created using the descent gradient methodology.

The authors showed that the median level of FC in this cohort was 125 *μ*g/g. LS (calculated in WL SBCE review) correlation with FC levels was *r* = 0.490 (*P* = 0.01), while for BM review and LS correlation with FC was *r* = 0.472 (*P* = 0.013), and the authors concluded that blue mode did not perform better than white light in calculating Lewis score [22].

### 4.2. Automatic Detection of Small-Bowel Mucosa

The new Data Recorder (DR3) by Given Imaging Ltd, Yokneam, Israel, not only stores the capsule's incoming images but also analyzes them in real time to control the capsule capture rate of images at an adaptive frame rate. When DR3 recognizes that the capsule is virtually stationary, it sets the image capture rate to 4 frames per second. When the DR3 recognizes that the capsule is in motion, it sets the image capture rate to 35 frames per second. A recent study tested the reliability of the automatic detection of the small bowel (SB) mucosa and the subsequent alert for booster ingestion by the Data Recorder 3 (DR3) of the second-generation colon capsule endoscopy (CCE-2). 120 consecutive cases of CCE-2 were analyzed for proper DR3 automatic detection of the capsule entering the SB. The DR3 correctly identified the proper time for ingestion of the laxative (booster) in 118 of 120 cases, corresponding to a sensitivity of 98.3% (95% CI, 97%–100%). The median time difference between DR3 automatic SB detection to the observed entrance of the capsule into the SB was 3 minutes 30 seconds (interquartile range 2 minutes 35 seconds to 5 minutes 57 seconds) [23]. 

### 4.3. Gastric Emptying

Another study was conducted to determine whether the use of an external real-time viewer could reduce delays caused by delayed gastric emptying of the capsule or delayed intestinal transit and also improve the rate of positive findings. The authors examined 100 procedures in the real viewer group and 100 control procedures in the age matched. In the viewer group, additional water intake (22 cases) and/or administration of metoclopramide (26 cases) were required. Endoscopic-assisted duodenal placement of the capsule was required in three cases. Overall one-third (*n* = 33) of cases required viewer prompted interventions. The completion rate (86% versus 66%, *P* = 0.002) and the rate of positive findings (80% versus 67%, *P* = 0.04) were significantly higher in the viewer group compared to the no viewer group [24]. 

## 5. Novel Indications

### 5.1. Surgery Using Intraoperative Real-Time Capsule Endoscopy

Identifying the exact site of small bowel hemorrhage is often difficult, thus complicating surgical treatment. A recent report was published on two cases of small bowel bleeding lesions that were successfully managed by intraoperative real-time capsule endoscopy. The authors developed a double lumen tube similar to, but thinner and longer than, the Miller-Abbott tube and insert the tube nasally, 3 or 4 days preoperatively, such that its balloon tip reaches the anus by the operative day. During surgery, the endoscopic capsule is connected to the balloon tip of the tube that protrudes from the anus. Capsule endoscopic images are displayed in a real-time video format. Minimally invasive surgery was successfully performed in both patients. The authors concluded that combined use of capsule endoscopy and the tube facilitates management of bleeding lesions in the small bowel [25].

### 5.2. Tumor Recognition

A recent paper addresses the automatic recognition of tumor for SBCE images. Extensive experiments validate that the proposed computer aided diagnosis system achieves a promising tumor recognition accuracy of 92.4% in SBCE [26].

### 5.3. Three-Dimensional Image Reconstruction in Capsule Endoscopy

A new software approach to approximate a 3D representation of digestive tract surface utilizing current SBCE technology has been tested. The authors showed promising results for polypoid structures and angioectasias [27]. 

### 5.4. Capsule Endomicroscopy

A study examined a new pill-sized endomicroscopy has been developed that enables 3D imaging of the esophagus in microscopic detail. The device uses optical frequency domain imaging technology (using infrared light) to provide architectural cross-sections of the esophagus, which can then be reconstructed into a 3D view of the length of the esophagus. The device was tested in 13 individuals (seven healthy volunteers and six patients with Barrett esophagus), with distinct differences in esophageal architecture observed between the two groups [28].

## 6. Conclusion

The major indication for SBCE is small bowel imaging specially in obscure gastrointestinal bleeding. The application of FICE could improve the characterization of angiodysplastic and vascular lesions and erosions or ulcers in small bowel lesions. We will see in the near future new capsule devices to enable targeted drug administration or even direct hemostatic therapy. Nevertheless, new capsule devices may improve both, polyp detection, and characterization rates. Integration of virtual chromoendoscopy techniques, like FICE will further improve image resolution and will help to better characterizes small bowel lesions.
